# Spots, stripes, and strains: a case report of multidrug-resistant *Salmonella* Newport in exotic felids

**DOI:** 10.1128/asmcr.00098-25

**Published:** 2025-11-10

**Authors:** Brianna L. S. Stenger, Daniel R. Evans, Sarah J. Gefroh, Alyssa Breuer, Heidi L. Pecoraro, Kelli J. Maddock

**Affiliations:** 1 Veterinary Diagnostic Laboratory, North Dakota State University 3323, Fargo, North Dakota, USA; 2 North Dakota Health and Human Services 1749 , Bismarck, North Dakota, USA; 3 Minnesota Department of Health 11055, St. Paul, Minnesota, USA; 4 Dakota Veterinary Hospital of Wahpeton, Wahpeton, North Dakota, USA; Pattern Bioscience, Austin, Texas, USA

**Keywords:** animals, exotic, antimicrobial suscetibility, *Salmonella*

## Abstract

**Background:**

Shedding of *Salmonella* is not unusual in captive exotic animals; however, few cases link *Salmonella* to chronic disease or death of exotic felids.

**Case Summary:**

We report a multidrug-resistant (MDR) *Salmonella enterica* serovar Newport, persisting strain REPJJP01, isolated from exotic felids from two separate regional zoos and potentially contributing to the death of a snow leopard (*Panthera uncia*) and a Pallas’s cat (*Otocolobus manul*). REPJJP01 strains of *S*. Newport are a recurring cause of human enteric disease outbreaks associated with travel to Mexico and beef products.

**Conclusion:**

This case highlights the value of genotypic and phenotypic techniques to identify shared and potentially MDR strains of *Salmonella* from human and animal hosts. Importantly, these cases expand the host range of *S*. Newport persisting strain REPJJP01.

## INTRODUCTION

Salmonellosis in humans and animals represents a significant One Health problem. *Salmonella* can be spread between species, and individuals (humans or animals) may be asymptomatic carriers and spread the bacterium to susceptible individuals. In addition, multidrug-resistant (MDR) strains are becoming more common. Exotic animals are known to shed *Salmonella*, often without showing symptoms, but evidence linking it to chronic disease or death in felids is limited (1–6). Raw meat fed to the animals is often suspected as the source (1–4), posing an occupational risk to the caretakers and a biorisk to other animals that may have a worrisome conservation status.

Here, we present the first cases of MDR *Salmonella enterica* serovar Newport persisting strain REPJJP01 in captive snow leopards (*Panthera uncia*) and a Pallas’s cat (*Otocolobus manul*). This case report was prepared following CARE Guidelines (7).

## CASE PRESENTATION

**Fig 1:**
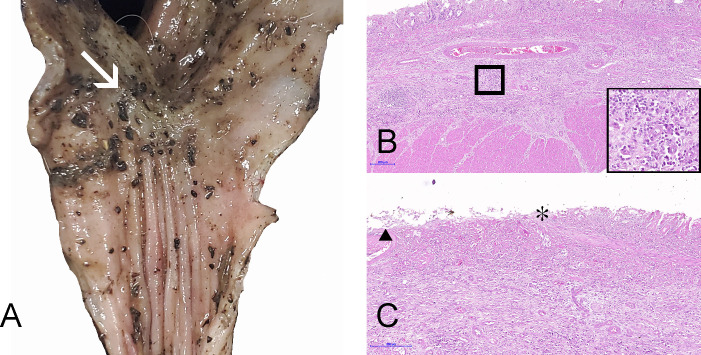
Snow leopard with colonic salmonellosis. (**A**) Gross image of the stricture. (**B**) Photomicrograph of inflammation expanding all layers of the colon, H&E 60×. Inset highlights lymphocytes and plasma cells, H&E 400×. (**C**) Photomicrograph depicts mucosal ulceration and erosion on the left and right sides of the asterisk, respectively; black triangle marks an intravascular fibrin thrombus, H&E 100×.

In March of 2022, a captive 8-year-old female snow leopard presented to the attending zoo veterinarian with more than a year of intermittent diarrhea and a 4-month history of periods of inappetence. Previous dental concerns and repetitive licking behaviors prompted sedation and examination. No significant lesions were noted in the mouth, but a granular material was observed in the colon by X-ray of the cranial abdomen, prompting a warm water and mineral enema. The snow leopard died several hours after the procedure, and a necropsy was performed by the attending zoo veterinarian. At necropsy, internal organs were grossly normal except for the colon, where multiple ulcerations were observed and a segment of the descending colon was abnormally small with a 0.5 cm colonic stricture (Fig. 1A). Local lymph nodes also appeared enlarged. Fecal material as well as fresh and fixed intestinal tissue and lymph nodes were submitted to the laboratory for further investigation.

Feline panleukopenia virus PCR, fecal flotation for parasites, and enteric culture on the intestines were performed. No feline panleukopenia virus or parasites were detected. Enteric culture was performed by plating the mucosal surface of the intestinal tissue directly to sheep’s blood and Tergitol 7 with triphenyltetrazolium chloride (Tergitol 7 TTC) agars and incubated aerobically at 35°C in ambient conditions for 24 h. A small piece (approximately 1 cm × 1 cm) of intestine was inoculated into tetrathionate broth and incubated in the same conditions. The intestines were also directly plated to Campy CVA (cefoperazone, vancomycin, and amphotericin B) agar and incubated at 42°C in microaerophilic conditions (CampyGen) for 72 h. At 24 h, no *Salmonella* or other enteric pathogens were observed. After 24 h, tetrathionate broth was subcultured to Tergitol 7 TTC and XLT-4 (Xylose-Lysine-Tergitol 4) agar and incubated at 35°C in ambient conditions for 24 h. Red colonies on Tergitol 7 TTC and black colonies on XLT4 were isolated and identified as *Salmonella* species by matrix-assisted laser desorption ionization time-of-flight mass spectrometry (Bruker Biotyper, Bruker Daltonics, Billerica, MA, USA). A single representative colony was selected for stereotyping. The National Veterinary Services Laboratory (NVSL; Ames, IA, USA) performed serotyping (8) and confirmed the isolate to be *S*. Newport.

On microscopic examination of the large intestine, the mucosa was extensively ulcerated, while the submucosa was expanded by fibrin deposition, necrotic debris, and mixed inflammatory cells. Lymphocytes and plasma cells multifocally invaded vascular walls of the submucosa and tunica muscularis (Fig. 1B). In addition, there were frequent intravascular fibrin thrombi. In the adjacent intact mucosa, there were sloughed cells into the cryptal lumens, multifocally, along with occasional crypt dilatation and necrotic debris. These findings are consistent with ulcerative colitis (Fig. 1C), which in people may be autoimmune in nature but has also been associated with *Salmonella* infection in both humans and animals.

A fecal sample from the asymptomatic enclosure mate was submitted for enteric culture as described above, and *S*. Newport was also isolated. The surviving snow leopard was treated with enrofloxacin (136 mg tablet) by mouth once daily for 7 days.

Initial whole-genome sequencing (WGS, DNA Prep, Illumina) of the cage mate isolates revealed identical *S*. Newport with multiple antimicrobial resistance (AMR) genes, prompting a retrospective review of previous exotic felid cases received at the laboratory. We identified another NVSL-serotyped *S*. Newport strain from a male Pallas’s cat (*O. manual*) housed in a second regional zoo the prior year (July 2021). This isolate was also further characterized as described herein.

To match genotype with phenotype, antimicrobial susceptibility testing (AST) was performed by Vitek2 Compact using AST-GN98 cards (bioMérieux, INC., Durham, NC, USA), COMPGN1F (enrofloxacin, Sensititre), Etest (azithromycin and ceftriaxone, bioMérieux, INC.), and disk diffusion (meropenem, BBL Sensi-Discs, BD, Franklin Lakes, NJ, USA) to the manufacturer’s instructions with quality control. For translatability to human isolates, Clinical and Laboratory Standards Institute (CLSI) M100 35th edition (9) human interpretive criteria for *Salmonella* and *Shigella* were used for interpretation of all AST, with the exception of enrofloxacin (Table 1). Enrofloxacin interpretations were derived from canine Enterobacterales clinical breakpoints in CLSI VET01S 7th edition (10), chosen because they were recently revised (11) to reflect both epidemiological cutoff and pharmacokinetic/pharmacodynamic considerations. Minimal inhibitory concentration (MIC) results were identical for all three isolates and meropenem zone diameters ranged from 30 to 32 mm (Table 2).

**TABLE 1:** CLSI clinical breakpoints for *Salmonella* and *Shigella* (CLSI M100 35th edition) and enrofloxacin canine Enterobacterales (CLSI VET01S 7th edition)

Antimicrobial	Minimal inhibitory concentration (MIC) clinical breakpoints (µg/mL)			
	Susceptible	Susceptible, dose dependent	Intermediate	Resistant
Ampicillin	≤8	–^*a*^	16	≥32
Ceftriaxone	≤1	–	2	≥4
Chloramphenicol	≤8	–	16	≥32
Ciprofloxacin	≤0.06	–	0.12–0.5	≥1
Doxycycline	≤4	–	8	≥16
Enrofloxacin	≤0.06	0.12–0.25	–	≥0.5
Imipenem	≤1	–	2	≥4
Azithromycin	≤16	–	–	≥32
Trimethoprim-sulfamethoxazole	≤2/38	–	–	≥4/76
Disk diffusion clinical breakpoints (mm)				
Meropenem	≥23	_	20-22	≤19

^*a*^ –, not available.

**TABLE 2:** Summarized antimicrobial susceptibility testing results and resistance genes detected^*a*^

Antimicrobial	MIC (µg/mL)	ZD (mm)	Interpretation	Resistance genes	Drug class
Ampicillin	≥32	–^*b*^	R	*blaCARB-2*	β-lactams
Ceftriaxone	0.06	–	S	*–*	Cephems
Chloramphenicol	≥64	–	R	*floR*	Phenicols
Ciprofloxacin	0.5	–	I	*qnrA1*	Fluoroquinolones
Doxycycline	≥16	–	R	*tet(A*)	Tetracyclines
Enrofloxacin	0.5	–	R	*qnrA1*	Fluoroquinolones
Imipenem	≤0.25	–	S	*–*	Carbapenems
Meropenem	–	30-32	S	*–*	Carbapenems
Azithromycin	64	–	R	*mph(A*)	Macrolides
Trimethoprim-sulfamethoxazole	≥320	–	R	*dfrA1* *sul1*	Trimethoprim, sulfonamides

^*a*^ Human clinical breakpoints (CLSI M100 35th ed.) used to determine antimicrobial susceptibility test results, with the exception of enrofloxacin, which was based on (CLSI VET01S 7th ed.) canine breakpoints. Results were identical for all three isolates, with the exception of meropenem. I, intermediate; MIC, minimal inhibitory concentration; mm, millimeter; R, resistant; S, susceptible; ZD, zone diameter.

^*b*^ –, not available.

All three *Salmonella* isolates were characterized by short- and long-read WGS and AST. Isolated colonies were placed into 200 µL of nuclease-free water and DNA/RNA extracted (KingFisher Flex, Thermo Fisher; MagMAX CORE, Applied Biosystems) according to the manufacturers’ instructions. For short-read (iSeq 100, Illumina) and long-read (MinION, Oxford Nanopore Technologies [ONT]) WGS, barcoded libraries were prepared using half-reagent volumes (12) (DNA Prep kit; Illumina) and full volume reagents (SQK-RBK004; ONT), respectively.

Quality control, species identification, short-read assembly, and quality check were performed with FastQC v0.11.8, Kraken2 v2.0.8, Shovill v1.1.0, and QUAST v0.5.2, respectively. Hybrid genomes were assembled with Unicycler v0.5.1, annotated with Bakta v1.9.4, core gene alignment constructed with Panaroo v1.5.0, and phylogenetic tree (short-read core genomes only) with IQTree2 v2.3.6. Single-nucleotide polymorphisms (SNPs) were identified from core gene alignments using snp-dists v0.8.2. Plasmid contigs were identified from hybrid-assembled genomes using mob-typer v3.1.9. AMR genes present on a shared plasmid were identified using AMRFinderPlus v4.0.19. Sequences of core plasmid genes were then wrapped into a reference database using ABRicate v1.0.1, which was then used to query their presence in all short-read assemblies.

**Fig 2:**
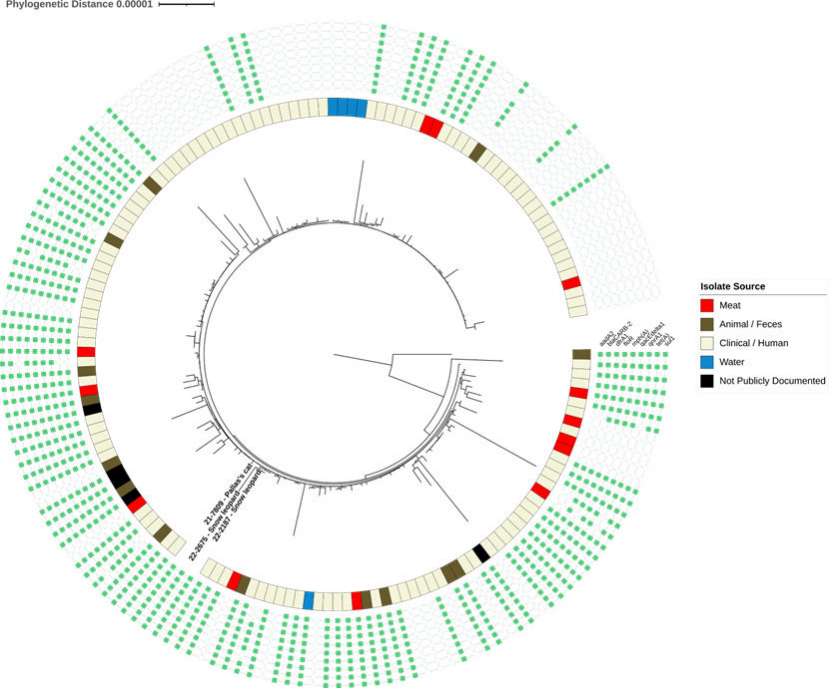
Maximum likelihood phylogeny constructed from 4,421 core genes of *Salmonella enterica* genomes from exotic felids and publicly accessible contextual data. Felid genomes are labeled by specimen ID number and species. Internal color strip denotes the specimen source, as documented in the NCBI Pathogen Detection database. Array of green squares denotes the presence or absence of AMR genes present on the shared IncR plasmid that were identified in each genome. Every genome displayed in the tree had an average sequence coverage greater than 30×.

All three *S*. Newport genomes were compared to public genomic data available through NCBI, which assigned them to the same Pathogen Detection cluster (PDS000007781.1237) that includes the MDR persistent strain, REPJJP01. Phylogenetic and SNP analyses of felid isolate genomes and contextual genomes identified from the same subclade resolved by the NCBI SNP Tree Viewer tool (*n* = 158 genomes) confirmed the close genetic relatedness of felid isolate genomes to those from human clinical, meat, animal fecal, and environmental isolates (Fig. 2). Core sequences of those genomes, which were assembled and assessed for quality by the same described short-read methods, ranged in genetic similarity from 0 to 293 SNPs (median: 8 SNPs, interquartile range [IQR]: 4–18 SNPs), with short-read assemblies from felids ranging in similarity to contextual genomes from 0 to 260 SNPs (median: 9 SNPs, IQR: 5–64 SNPs).

Long-read sequencing and hybrid genome assembly of felid genomes resolved a circularized 47 kb plasmid that was shared by all three genomes. This plasmid carried nine AMR genes that encoded resistance to aminoglycosides (*aadA2*), beta-lactams (*blaCARB-2*), trimethoprim (*dfrA1*), fluoroquinolones (*qnrA1*), macrolides (*mph(A*)), phenicols (*floR*), quaternary ammonium compounds (*qacEdelta1*), sulfonamides (*sul1*), and tetracyclines (*tet(A*)) (Fig. 2). These AMR genes were only variably present among closely related contextual genomes.

## DISCUSSION

While *Salmonella* may be shed asymptomatically and the presence of these strains may be incidental for the cage mate and Pallas’s cat, we believe the intestinal stricture observed grossly and the histopathologic changes observed in the snow leopard were consistent with salmonellosis and likely contributed to the death of this animal. We did not receive a complete tissue set for this case, so we cannot conclusively rule out comorbidities.

For all strains in this case report, the phenotypic AST results were supported by the detection of corresponding plasmid-encoded AMR genes. REPJJP01 strains are MDR, limiting treatment options. First identified in 2016, REPJJP01 strains have been detected in humans residing in all 50 US states and have previously been associated with travel to Mexico and beef products (13). No human cases were epidemiologically linked as potential events of direct transmission to these isolates.

Treatment of asymptomatic *Salmonella* infections is not recommended due to the potential for prolonged shedding and risk of perpetuating AMR development. In support of this recommendation, our laboratory does not routinely perform AST on *Salmonella* isolates from enteric sources. Treatment of the asymptomatic snow leopard was elected due to the potential for death loss of this vulnerable species.

Potential sources of *Salmonella* in captive felids could be meat (1–4) or treat suppliers, caretakers (reverse zoonoses), or wild animals, such as birds or rodents with access to the zoo enclosures (1–4, 14–17). Unfortunately, no meat or treats contemporaneous to the time of suspected infection were available for testing. Workers were educated on appropriate biosecurity for moving between enclosures, proper handling of food, and handwashing procedures.

In conclusion, we report the *S*. Newport strain REPJJP01 in snow leopards and a Pallas’s cat, expanding the host range for this pathogen. We recommend continued characterization of *Salmonella* strains isolated from animals to help close epidemiological gaps.

## Data Availability

Whole-genome sequencing reads for isolates collected from felids are publicly available in NCBI under the BioProject PRJNA860199: SRX16349265, SRX20641647, SRX20641648, SRX20641649, SRX20646762, SRX20646763, SRX20646765, SRX20646766, SRX20646767, and SRX20646768.
